# Failure of early extubation among cases of coronavirus disease-19 respiratory failure: Case report and clinical experience

**DOI:** 10.1097/MD.0000000000020843

**Published:** 2020-07-02

**Authors:** Jingchen Zhang, Xujian He, Jia Hu, Tong Li

**Affiliations:** Department of Emergency, the First Affiliated Hospital, Zhejiang University, Hangzhou, China.

**Keywords:** coronavirus disease 19, extubation criteria, mechanical ventilation, reintubation

## Abstract

**Rationale:**

Extubation strategy for mechanically ventilated patients with Coronavirus Disease 19 is different from that for patients with other viral pneumonia. We reported 2 cases of Coronavirus Disease 19 receiving tracheal intubation twice during the hospitalization.

**Patient concerns:**

Two elderly patients with onset of fever and upper respiratory tract infection were confirmed as Coronavirus Disease 19, 1 of whom had chronic obstructive pulmonary disease previously. With active antiviral and noninvasive respiratory supportive therapy, there was no improvement, thus mechanical ventilation (MV) was adopted. Combining with symptomatic and supportive treatment, their oxygenation recovered and then extubation was carried out. However, 96 hours later, they underwent endotracheal intubation again due to their Coronavirus Disease 19 progression.

**Diagnosis:**

Critically ill Coronavirus Disease 19 requiring tracheal intubation owing to respiratory failure with lung.javascript.

**Interventions:**

Initial Strategy for respiratory failure included endotracheal intubation, MV, antiviral treatment and cortisol in both cases. When extubation criteria were satisfied, early discontinuation of MV was conducted, then rehabilitation exercise and nutritional support followed. However, 96 hours later, the disease progressed leading to respiratory failure again, thus reintubation was performed. Later, veno-venous extracorporeal membrane oxygenation was performed owing to aggravation of respiratory failure, assisted by prone position treatment and sputum drainage, then status became stable and stepped into recovery stage.

**Outcomes:**

Both patients underwent reintubation, and their MV time and Intensive care unit residence time were prolonged. Through prone position treatment, sputum drainage and awake extracorporeal membrane oxygenation strategy, patient has been transferred to rehabilitation unit in Case 1, and patient in Case 2 has been in recovery stage as well with stable pulmonary status and was expected to receive evaluation in recent future.

**Lessons:**

Course of Coronavirus Disease 19 is relatively longer, and failure rate of simple early extubation seemes higher. To reduce the likelihood of reintubation and iatrogenic injury, individualized assessment is recommended.

## 1 Introduction

Physiologically, the cases of Coronavirus Disease 19 (COVID-19) presented with bilateral diffuse alveolar injury accompanied with myxoid exudate.^[1]^ Relevant clinical manifestations included long course of disease, rapid progression of respiratory failure among critical patients, of whom most required mechanical ventilation (MV) for life support.^[2]^ We reported 2 critical patients with early discontinued MV through extubation, who were reintubated for MV 96 hours after the extubation. COVID-19 shows a clinical development process different from previous viral pneumonia. At present, little is known about occurrence and development of COVID-19, and the timing for the extubation is controversial.^[3,4]^ We provided the 2 patients who were intubated 96 hours after extubation.

## 2 Case presentation

**Figure 1:**
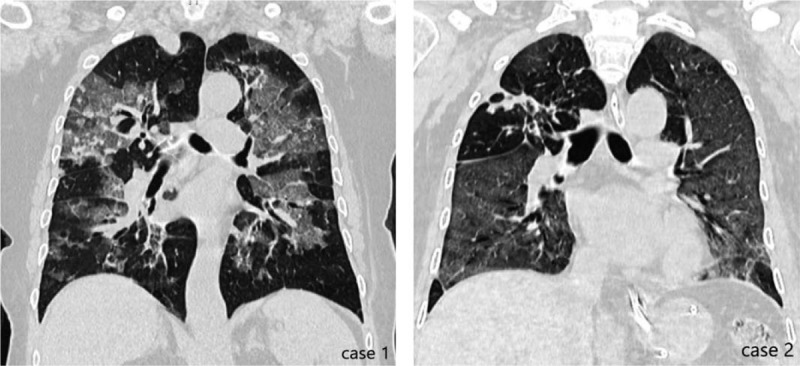
Chest CT scans of case 1 and case 2 when they entered ICU. CT = computed tomography, ICU = intensive care unit.

**Figure 2:**
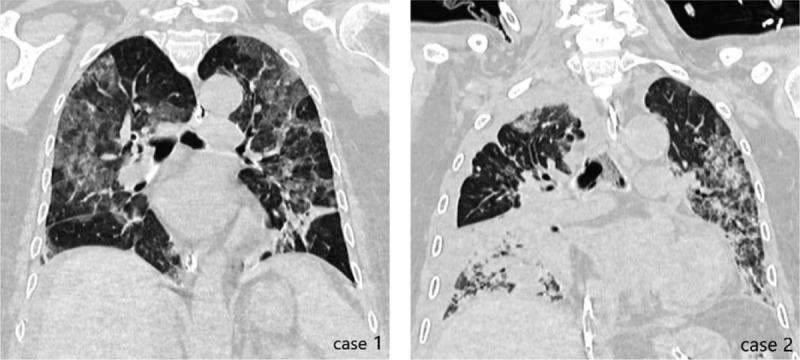
Chest CT scans of case 1 and case 2 when they were intubated for the first time. CT = computed tomography.

**Figure 3:**
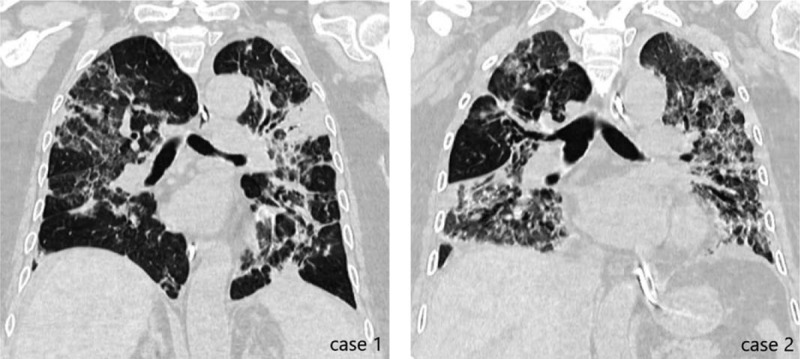
Chest CT scans of case 1 and case 2 when they were extubated for the first time. CT = computed tomography.

**Figure 4:**
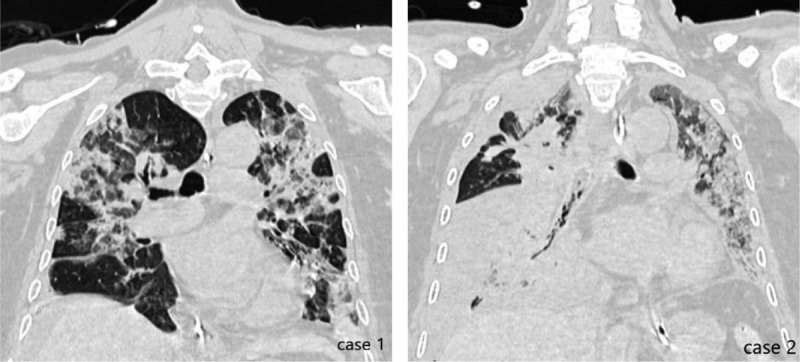
Chest CT scans of case 1 and case 2 when they were intubated for the second time, CT = computed tomography.

**Figure 5:**
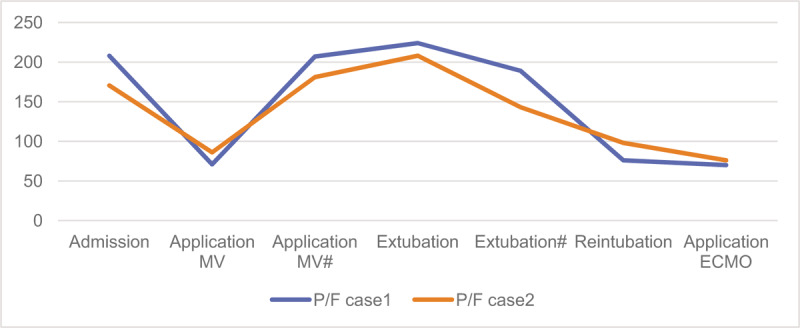
Clinical courses and outcomes in these 2 cases. # second day; MV = mechanical ventilation; P/F = oxygen permeance/fraction of inspiration oxygen.

In Case 1, a 74-year-old woman, were given MV for respiratory failure 9 days after COVID-19 confirmation (Figs. 1 and 2). After evaluating oxygenation, her condition met the criteria of extubation (listed below). The patient was extubated 4 days after MV, during which antiviral treatment and infection prevention based on her symptoms were also given (Fig. 3). After extubation, high flow nasal cannula was given for respiration assistance, and also, pulmonary function rehabilitation and nutritional support were provided. The chest X-ray examination showed a slow progress, followed by progressive decrease in oxygenation index, respiratory rate-oxygenation (ROX) index:^[5]^ 3.6 to 3.7, and then, according to the indications of tracheal intubation, the first criterion was satisfied, thus the patient was intubated again 96 hours after the first extubation (Fig. 4). On the next day, veno-venous extracorporeal membrane oxygenation (ECMO) was performed for her, flow rate: 60 ml/ (kg/min), airflow/blood flow: 0.8 to 1:1. On the third day, the patient was extubated and awaking ECMO was adopted. Nucleic acid test of SARS-CoV-2 virus became negative 5 days after the second intubation. After extubation, the patient could take food by himself. She is in good mental state and full of confidence in recovery. The patient has been transferred to the rehabilitation unit. Her clinical course is shown in Figure 5.

In Case 2, an 80-year-old man with a history of Chronic obstructive pulmonary disease, was admitted to our hospital after intubation for MV due to respiratory failure 5 days after COVID-19 confirmation (Figs. 1 and 2). After 11 days of treatment, after evaluating oxygenation, his condition met the criteria of extubation. the MV was discontinued (Fig. 3), and high flow nasal cannula was given for respiration assistance. Then the patient had delirium episodes without sputum excretion disturbance. Then oxygenation condition became worsened, ROX index: 3.5 to 3.7, accompanied with PCO_2_ > 50mm Hg, and the chest X-ray examination showed a progress. According to the first criterion of tracheal intubation, the patient was intubated again 120 hours after the first extubation (Fig. 4). On the 5th day, veno-venous ECMO was performed for him, flow rate: 60 mL/ (kg/min), airflow/blood flow: 0.8 to 1:1. Then the patient was extubated and given ECMO in an awakened state. Nucleic acid test of SARS-CoV-2 virus became negative 3 days after the second intubation. Dextrometomidine was given for slight sedation. The patient had a large amount of pleural effusion, which was treated with puncture and drainage, and gastrointestinal bleeding was found. Gastroscopy showed diffuse bleeding in the tail. Her clinical course was shown in Figure 5.

Extubation and intubation indicators

Extubation indicators (all the followings should be satisfied at the same time):

(1) having a conscious mind, able to follow relevant instructions;
(2) Sputum drainage frequency < 1/4 hour;
(3) spontaneous breathing pattern positive end-expiratory pressure ≤5 mm Hg, RR < 25 times/min, spontaneous breathing trial accomplished, PO2/FiO2 > 200;
(4) norepinephrine dose less than 0.5 μg/(kg × min), and lactic acid < 2mmol/L.

Intubation indicators (satisfying any 1 of the following criteria indicates the requirement of intubation):

(1) HFNC oxygen flow 40–50L/min, FiO2 100%, for a 2 h observation period, if the ROX index < 3.85 or SPO2 < 93% and RR > 30 times/min;
(2) disturbance of consciousness;
(3) malignant arrhythmia;
(4) severe shock (noradrenaline exceed 1 μg/(kg × min));
(5) acute respiratory acidosis (PH <7.25).

These indicators are standard guidelines of the First Affiliated Hospital of Zhejiang University, and they are also indicators for the treatment of COVID-19.

## 3 Discussion

Up to now, the mortality rate among COVID-19 patients in Hubei Province has been up to 1.4%, and the epidemic is spreading globally with time.^[6]^ According to the recent reports,^[7]^ old patients are more prone to be infected by COVID-19, especially for those with underlying diseases. At present, the pathophysiological process of critical patients is still to be fully understood. Through observation on the 35 critical patients treated in the center, 16 of whom required MV, as well as a comparison with previous viral pneumonia, we found that COVID-19 progressed faster and had a longer course. The patients in our center are mild cases and medical conditions are sufficient.^[8]^ So, we adopted the early extubation strategy. Total 5 patients were extubated in our center, among which 3 patients underwent tracheotomy and 2 patients received early intubation. In this report, 2 patients showed good respiratory and systemic conditions within 48 hours after extubation, and we believed that the extubation was successful. However, both patients received endotracheal intubation again and ECMO assisted therapy 96 hours after extubation.

The early extubation strategy was recommended by the first-line clinical experts in China. In this study, the 2 patients were all of old age, the medication regimen was based on relevant national guidelines, and the clinical manifestations were improved after MV Within 48 hours after extubation, the patient still showed a good oxygenation condition, good deglutition and good swallowing and movement coordination. The strategy was consistent with the thoughts for previous diagnosis and treatment of viral pneumonia. However, the 2 patients in our center, who strictly followed the extubation strategy,^[9]^ and were given HFNC for breathing assistance after the operation, with ROX index above 8 and breathing frequency less than 25 times, and felt good (The chest X-ray examination showed no obvious progress and the patients showed good response after extubation) in the first 48 hours, were intubated again due to respiratory failure 96 hours after extubation, and the conditions for intubation met relevant indicators strictly. We believed that this was mainly due to the continued progress of the primary disease and inadequate sputum drainage, thus the condition got worse. Subsequent treatment response proved this hypothesis in these 2 cases.

At present, the MV indicators for the removal of viral pneumonia are all based on clinical manifestations and auxiliary examinations, which are applicable for the short and self-limited course of conventional pneumonia. However, clinical manifestations of COVID-19 are different from those of conventional pneumonia, and so, new extubation indicators are required. In the clinical practice of our center, we found that extubation indicators are not only based on clinical manifestations and imaging findings, but also depends on viral test result conversion to negative, which should also be taken as an important reference.

We wish to remind relevant people to take the early extubation strategy cautiously and implement individualized extubation plan for COVID-19 patients with MV.

## Acknowledgments

The authors would like to thank all participants of the study, the nurses and clinical staff who are providing care for the patients.

## Author contributions

JC Zhang generated the idea of writing the case report and was the consultant in charge of the patient. JC Zhang and XJ He reviewed the case notes of the patient and wrote the original draft of the case presentation. J Hu and T Li significantly revised the original draft and added the conclusions and references. All authors contributed to the final version of the manuscript.
